# Exposome and Social Vulnerability: An Overview of the Literature Review

**DOI:** 10.3390/ijerph19063534

**Published:** 2022-03-16

**Authors:** Séverine Deguen, Mary Amuzu, Valentin Simoncic, Wahida Kihal-Talantikite

**Affiliations:** 1 Department of Social Epidemiology, INSERM, Institut Pierre Louis d’Epidémiologie et de Santé Publique (UMRS 1136), Sorbonne Universités, UPMC Univ Paris 06, 75646 Paris, France; 2 EHESP School of Public Health, 35043 Rennes, France; mary.amuzu@eleve.ehesp.fr; 3 LIVE UMR 7362 CNRS (Laboratoire Image Ville Environnement), University of Strasbourg, 67000 Strasbourg, France; valentin.simoncic@live-cnrs.unistra.fr (V.S.); wahida.kihal@live-cnrs.unistra.fr (W.K.-T.)

**Keywords:** exposome, neighborhood and environmental inequalities, environmental nuisances, social vulnerability, socioeconomic status

## Abstract

Background—The exposome concept refers to the totality of exposures from internal and external sources, including chemical and biological agents from conception throughout the lifetime. Exposome is also made up of psychosocial components such as socio-economic status (SES), which will focus on in this review. Despite exposures to the same environmental nuisances, individuals and groups are impacted differently. According to the literature, health inequalities exist among different socioeconomic groups, and SES may influence the association between environmental nuisances and health outcomes. However, the variation of this interaction across ages has rarely been studied. There is a need to adopt a life course approach to understand the history of diseases better. Objective—The main objective of this review is to document how SES could modify the association between environmental nuisances and health outcomes, across different ages, as a first crucial step introducing the emerged concept of social exposome. Methods—The PubMed database was searched from January 2010 to August 2021 for systematic reviews published in English addressing the interaction between SES, environmental nuisances, and health outcomes. Socio-economic indicators considered include education, level of income, neighborhood environment. Environmental nuisances considered many environment nuisances, mainly air pollution and noise. Results—Among 242 literature reviews identified, 11 of them address the question of the effect modification. Overall, our work reveals that environmental nuisances were mostly associated with poorer health outcomes and that SES modified this association, increasing the health risk among the poorest. Very interestingly, our work reports the existence of this interaction across different ages, including pregnancy, childhood, and adulthood, and for various environmental nuisances. Conclusion—In conclusion, our work confirms that we are not all equal to face environmental nuisances. The poorest are more vulnerable to the health effect of environmental nuisances. Policy decisions and interventions should target this high-risk population as a priority. Further investigations are needed to formalize the concept of social exposome more precisely and then communicate about it.

## 1. Introduction

Environmental nuisance refers to the unreasonable interference or likely interference with the environmental value caused by air, noise, water, soil pollution, and heat emissions and are harmful to the environment and the wellbeing of the local population [1]. Environmental nuisances are health determinants and influence health outcomes. Health determinants include physical, social, and economic environment and individual characteristics and behaviors [2].

Over the past decade, environmental exposures have become increasingly recognized as a risk factor underlying many health outcomes such as cardiovascular diseases, respiratory diseases, and neuropsychological diseases, leading to many premature deaths. For example, air pollution (both indoor air and outdoor air pollution) causes up to at least seven million premature deaths yearly. The deaths disproportionately affect marginalized populations of lower SES [3]. However, people are exposed daily to a multitude of factors simultaneously, including environmental nuisances, community resources, medical conditions, genetic influences, and behavior that may jointly affect their health status. In this context, the exposome concept has recently been put forward as a way of describing the totality of these lifetime human environmental exposures.

Exposome is a concept in Public Health introduced by Dr. Christopher Oscar Wild to the scientific community in 2005. Through this concept, he shared his vision for the need to develop a field that provided an environmental complement to the genome. He defined it as the totality of exposures from various internal and external sources from conception onward over a complete lifetime [4,5]. The concept of exposome is to complement the genome to better explain the causes of diseases [6]. According to siroux et al [7], there are three overlapping main domains of this concept; the internal sources reflect the biological response to the external exposome, then the external sources, classified as general external and specific external. The general external sources include social capital and education, and the specific external sources include chemical contaminants, environmental pollutants, and lifestyle factors.

The exposome concept considers the lifelong environmental condition from the conception period, which accumulates from the sensitive period of conception to old age and can impact health status [8]. However, to date, most environmental research investigates the environmental condition at a single exposure window (e.g., pregnancy, infancy, childhood, adulthood, and finally old age) and does not cover the environmental condition of lifelong.

Life-course approach has become a guiding framework for researchers in health, policymakers, and health practitioners. This had led to research in different fields such as chronic diseases, epidemiology, biology, genetics, economics, sociology, etc., into a cohesive conceptual health model better to understand the history of diseases [9]. The life course theory holds that individual and population health is influenced by the timing and sequence of biological, social, and historical events and experiences. For this review, several main life stages will be considered; Conception and pregnancy (pregnant women), Infancy and childhood, Adulthood, and finally, Old age [10]

‘’Inequalities in health, thus refers to the composite measure of the differences in health status across individuals or groups in a population’’ [11] while social inequalities in health arise from differences in Socioeconomic Status (SES) and influence vulnerability to diseases. Evidence has accumulated, establishing a correlation between social inequalities and health outcomes [12,13]. Several public health studies in social epidemiology investigated how socioeconomic characteristics increase health inequalities. Scientific literature advanced the hypothesis by which environmental exposures might be combined to the role of social determinants.

To document how environmental exposures might interact with social determinants to contribute to health inequalities, the following hypotheses were advanced:

The first hypothesis is the differential exposure: deprived populations or those living in a deprived area are more likely to be exposed to a higher number of environmental nuisances or a higher level of environmental exposure.

The second hypothesis, the vulnerability differential suggests that deprived population or those living in a deprived area is particularly vulnerable to health effects of environmental exposures.

For example, the health consequences of air pollutants exposure during the pregnancy stage can be modified by her SES, which influences her ability to afford and access healthcare. The neighborhood environment where pregnant women live also plays a role [14]. Further studies have shown evidence of the impact of social and built neighborhood characteristics on health either through health promotion or serving as a barrier to health promotion [15].

Differential vulnerability refers to the potential negative effects caused by external factors on human health. These factors could be natural or man-made or disease outbreaks and vary among communities. Three dimensions underline vulnerability which are exposure, susceptibility, the capacity to respond through coping and adaptability, and the ability to recover. The notion of risk with vulnerability implies that everyone is potentially vulnerable or at risk of developing health problems, but its effects vary from person, community, and system. Most studies suggest that vulnerability is increased for individuals or groups with the lowest SES, and this influences their health outcomes and their capacity to respond [16].

Many studies investigate the environmental inequalities of one part of the general external exposome such as air pollution, noise, or green space. As already mentioned, many studies state that environmental exposure may contribute to health inequality through a different pathway. However, even the external exposome includes the social dimension as one health determinant, today, it remains unclear whether, over its life, all parts of the external exposome are similar or not according to the SES stratum. It is what Robinson and al. recently explored restricted to the pregnant women population [17]. Several methods assess socio-spatial differentiation or disparities through various independent variables and dimensions. Some papers propose a method to assess differential exposure by socio-demographic groups in intra-urban spaces as geo-demographic analysis. This constitutes a multidimensional analysis of social conditions, departing from the most detailed spatial disaggregation possible such as census tracts, postal codes, street blocks, or even households or individuals [18,19].

Given the recent literature, it seems necessary to summarize the scientific evidence to identify whether socio-economic status could modify the health impacts of environmental nuisances across different age groups. We hypothesize that low SES combined with cumulative adverse environmental conditions (exposure environment, green space, and built environment) may influence the health status of people at each stage of life from conception throughout the lifetime.

In this context, we perform a scoping review structured in three consecutive steps:

Firstly, a summary of the studies by extracting several information related to external exposome, social vulnerability, and health consequences;

Secondly, an analysis of the underlying concepts of the external exposome and social vulnerability defined by the different studies;

Finally, a combination of evidence addresses the question of whether or not the health consequences from environmental nuisances may be modified by SES at each stage of life, resulting in the definition of several exposomes according to the socioeconomic status. It tends to appear in the scientific community called the social exposome.

## 2. Material and Methods

### 2.1. Search Strategy

A scoping review was conducted using the PubMed platform providing access to the MEDLINE databases, among articles published between January 2010 and August 2021. The search strategy followed the PRISMA (Preferred Reporting Items for Systematic reviews and Meta-Analyses) guidelines and was performed with the following equation:

(noise [title] OR nuisance [title] OR nuisances [Title] OR heat [title] OR NO2 [title] OR PM [Title] OR environment [title] OR pollutants [Title] OR air pollution [Title]) and (deprivation [Title] OR socio-economic [Title] OR socioeconomic [Title] OR socioeconomics [Title] OR inequality [Title] OR inequalities [Title] OR contextual [Title] OR disadvantage [Title] OR disadvantages [Title] OR disadvantaged [Title] OR advantage [Title] OR advantages [Title] OR advantaged [Title] OR income [Title] OR employment [Title] OR unemployment [Title] OR neighborhood [Title] OR neighborhood [Title] OR lifestyle [Title] OR socio-occupational [Title] OR insurance [Title] OR educational [Title] OR social [Title] OR healthcare [Title] OR social [Title] OR susceptibility [Title] OR vulnerability [Title]).

Only literature reviews were considered in our work as many have already been published in this environmental health area.

### 2.2. Studies Selection Strategy

The criteria for inclusion were peer-reviewed articles written in English, with no restrictions on geographic location and human studies. We limited our scoping review to systematic reviews that were already published.

A summary of the search strategy used is as follows:

(i) The database PubMed, with the search date from January 2010 to August 2021 was used. This time frame was used to work with the most recent guidelines and policy framework regarding environmental issues as countries keep on changing their policies over time. This was performed to achieve homogeneity among the studies.
(ii) The titles of articles and their abstracts were reviewed based on the inclusion and exclusion criteria.
(iii) As a classical step, the reference list of the selected articles was also screened for possible additional articles.
(iv) The initial search yielded 242 results. Thirty systematic review articles were retained to document the two different mechanisms known to explain the relation between socioeconomic status, nuisance, and health; namely, it is the differential of exposure and the social differential of vulnerability. This work aims to document only the second mechanism, as the first one has been already published.
(v) Systematic reviews of 12 articles were retained solely for the work on exposome and social vulnerability.
(vi) During the data extraction, one article was excluded as there is no quantitative result.

**Figure 1:**
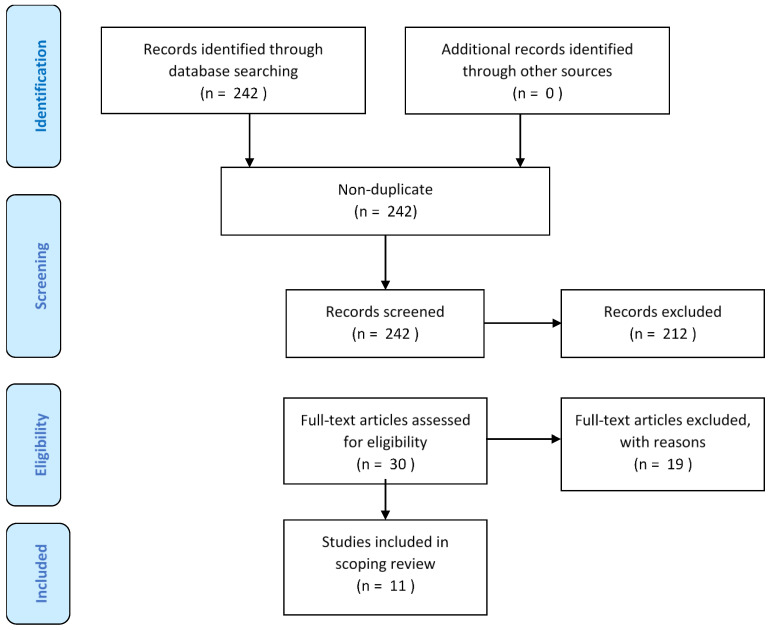
Flowchart of study selection [20]. From: Moher D, Liberati A, Tetzlaff J, Altman DG, The PRISMA Group (2009). Preferred Reporting Items for Systematic Reviews and Meta-Analyses: The PRISMA Statement. PLoS Med 6(7): e1000097. doi:10.1371/journal.pmed1000097.

Figure 1 summarizes the different steps of the selection process, in line with guidelines of Preferred Reporting Items for Systematic Review or Meta-analysis (PRISMA).

### 2.3. Study Selection

The studies were selected by one author, myself, and a second (WKT and SD) verified the selection. Any discrepancies in the selection were discussed by the team. The screening process involved the evaluation of the titles and abstracts of the retrieved studies, in accordance with the inclusion criteria. The full text of the articles retrieved was assessed. Where there were disagreements, they were resolved through discussions till the team reached a consensus.

### 2.4. Data Extraction

The data from the included studies were extracted using an adapted piloted Excel form. The excel instrument was adjusted during the review. For each study, we extracted and reported in the table the following information: General information (first author’s name, country of origin, and date of study), main study characteristics (number of studies included, population group, main findings), outcome measures (definition, outcomes classification, and source).

Synthesis of the results—The present study is a scoping review, so there was no statistical analysis planned or executed. Only descriptive synthesis was conducted.

## 3. Results

### 3.1. Studies Selected for Scoping Review

The preliminary search yielded 242 studies for the literature review published between 2010/2011-2021. Based on titles and abstracts, 212 studies were excluded. Reasons for exclusion were: (i) studies without socio-economic indicators (ii) animal studies (iii) Duplicates. The 30 articles were assessed in detail reading the full texts, 11 articles were retained as they dealt with social exposome.

### 3.2. Overall Description

Among the literature reviews, one was published in 2014, three in 2015, one in 2016, two in 2017, zero in 2018, two in 2019, one in 2020, and one in 2021. In total, seven (66%) of the literature review studies used descriptive analysis and did not perform quantitative analysis and four (33%) of the literature review studies performed meta-analysis. All 11 studies assessed multiple regions: Europe, the Americas, and Asia. However, one of the studies focused on Ireland. In all, nine systematic reviews examined Europe, seven, America, four, North America, and three Asia. The average number of individual studies included in the systematic reviews was eighty (80) studies. The lowest study had 14 included studies, and the highest number of included studies was 146.

#### 3.2.1. Social Conditions

The systematic review conducted by Paterson et al. included 15 studies and assessed people of low SES and urban dwellers as vulnerable groups. There is increased vulnerability to heat stress among urban dwellers due to overcrowding in homes, inability to afford air conditioning, poor general health, lifestyle risk factors, and living in densely populated areas.

The authors also identified outdoor workers as a vulnerable population [21]; here, we considered the outdoor workers as a specific social group. They were regarded as heat vulnerable. According to [21]., outdoor workers are at risk of six out of seven climate-related occupational hazards, such as increased ambient temperature, air pollution, ultraviolet radiation, extreme weather, vector-borne disease, and other biological hazards [21].

Benmarhnia et al. assessed 61 studies in their review and defined vulnerable groups based on individual SES. The authors identified the following as vulnerable groups, individuals with low SES, the population living in high-density areas, and unmarried individuals (to account for isolation) [22].

Fuller et al. assessed 30 articles in their review and examined vulnerable groups based on psychosocial stress and material resources as indicators of SES [23].

In the recent review, Mathiarasan et al. focused on children with low SES as a vulnerable group. The study assessed children’s health and their vulnerability through social determinants, including lack of access to resources and treatments, social discrimination, and low level of education [3].

Hibbert et al. also examined children with low SES as a vulnerable population. The authors found that the assessment of social vulnerability was based on economic measures including wealth, income, disposable income, or an index such as socioeconomic status (SES) or position (SEP), or poverty [24].

Alderton et al. included 14 studies in their review and also assessed low SES children as a vulnerable group [14]. The authors describe that the children from neighborhoods with poor housing quality, density, and inadequate nature or public open spaces in the neighborhood were considered as more vulnerable as compared to children from high-income neighborhoods.

Wong et al., examined the impact of physical exposures on the development and response to chronic diseases in later life and the influence the vulnerability of the individual including such as income, food security, education, and access to comprehensive health care [25]. Gelormino et al. included 23 articles in their study that assessed the relationship between the built environment and the health inequalities [26] among people in general. According to the authors, social variability was measured at the contextual level (neighborhood or country level) using various socioeconomic variables including poverty, income, education, employment, health insurance, and composite index such as Townsend. Schule et al., included 33 studies in the review to investigate how Both socioeconomic neighborhood characteristics and factors of the built environment play an important role for health among people in general. The authors described that social vulnerability was assessed using a socio-economic index, education level, or income [27]. Burte et al., included 25 studies in their review and explored children and adults as their population group. The social vulnerability was investigated using socioeconomic status families [28]. Erickson et al., examined fetal–placenta development. This paper explored the social vulnerability of pregnant women as to air pollutants and subsequent pregnancy outcomes [29]. All the studies included in this systematic review identified infants and children, adulthood, the elderly, and people in general such as people from low SES and people in specific occupations as vulnerable groups.

As we describe below, the concept of “social vulnerability” differs between studies according to the type of data used. Some authors used individual characteristics such as the income of unmarried individuals; others used neighborhood characteristics such as density and inadequate nature or public open spaces in the neighborhood. Some authors used home characters including overcrowding in homes or poor housing quality.

#### 3.2.2. Environmental Nuisances

The pollutants reviewed in the articles selected under the inclusion criteria were: Nitrogen dioxide (NO2), Nitrogen oxides (NO), Sulfur dioxide (SO2), Carbon monoxide (CO), Ozone (O3), Ethanol, Black carbon, p353-nonylphenol, Chlorfenvinphos, Chlorpyrifos, Nicotine, Organophosphates, smoke, polycyclic aromatic hydrocarbons (PAHs) and particulate matter with an inhalable coarse fraction (PM10, 2.5–10 µm), the fine respirable fraction (PM 2.5 ≤ 2.5 µm) and the ultrafine fraction (UFP, ≤0.1 µm). Nine out of the eleven studies examined environmental pollutants in the neighborhood environment and the remaining two studies analyzed exposures related to heat–health vulnerability and mortality.

#### 3.2.3. The Social Exposome

Exposome looks at all the exposures an individual is subjected to during the lifetime and how those exposures affect health outcomes. All the 11 articles (Table 1) in this literature review did not explicitly mention the term exposome, however, they all examined at least one pollutant and a particular life stage. Different life stages assessed in the articles as mentioned earlier included conception, infancy, childhood, adulthood, and old people/the elderly. However, only a few studies focused on old people.

The exposures examined by Mathiarasan et al. include environmental injustice on the health outcomes of children. The authors explored air pollution and climate change and its interaction with SES and how it affects the health of children [3]. Paterson et al. and Benmarhnia et al. explored the association between high temperatures and adverse birth outcomes of preterm birth, low birth weight and stillbirths and the elderly ages >65 and >75 years and socio-economic factors such as education, ethnicity, income, or social isolation [21,22]. Both authors mentioned above examined SES at the individual level and how it influences responsiveness to environmental exposures. The authors examined the vulnerability of poor people to heat and the subsequent reduction in their adaptive capacity. Paterson et al. explored the indirect impact of environmental exposures on human health from the perspective of food systems that is the decline of some species, food production, and soil erosion, which affects the quality of life through malnutrition [21]. Other studies examined the effect of a neighborhood-built environment on an individual during different life stages and how it is influenced by the SES [3,14,23,25,26,29]. Burte et al. assessed long-term exposure to air pollution and incident asthma among children and adults from low socio-economic backgrounds and possible susceptibility factors [28]. Hibbert et al. examined the impact of SES on chemical stressors. The study combined non-chemical stressors and chemical stressors. The non–chemical stressors were organized into topic areas which include acculturation, adverse childhood experiences (ACE), economic, education, family dynamics, food, green space, neighborhood, social stress, urbanicity, violence, and ‘other’ (other included several variables that were considered in the individual studies but did not fall under any of the categories [24].

In summary, to answer the research question “How the socioeconomic status modifies the environmental exposure health consequences at different stages of life?”, we considered the different important life stages from conception through to old age. We have analyzed the environmental health consequences and more precisely the effect modification of this relation by the socioeconomic status/conditions measured at individual or contextual level. All results are summarized in the following table. It shows the diversity of health consequences related to various environmental exposure; association modified by the socioeconomic status. These results are in favor of the existence of a social exposome.

## 4. Discussion

### 4.1. Main Findings

This review has provided a starting point to explore the exposome and social vulnerability of an individual/or a population and its subsequent health outcomes. The review shows the concept of social exposome being documented for different life stages throughout the studies included in this literature review although the exact terminology of exposome was not referred to in the included studies. More than 6 out of the 11 studies for this literature review, assessed pregnancy and childhood accounting for more than 50% of the studies which were included. However, there was not anyone document that looked at all the life stages is from conception through to old age. Only a few studies provided evidence on the older population as only two 2 studies reviewed the effect of exposome on this vulnerable group. Three 3 studies had a contrary opinion; (1) overtime one study found higher difficulties in children whose mothers were highly educated but had no access to green space (private garden) at home, (2) women of higher SES who used assisted reproductive services for childbirth recorded high adverse birth outcomes and (3) finally noise pollution negatively affected persons from high SES in their neighborhood environment in a study conducted in France.

This review provides an overview of the evidence of vulnerability and exposome using a life course approach from conception through to old age thus providing a starting point for increasing interest. The strength of this study is that the search strategy was not limited to specific health outcomes or age groups (which was the case in the literature reviewed), for instance, one of the studies focused on incident asthma and children and also during the literature search one of the studies focused on a particular organ, for instance, the skin or kidney with regards to exposome. This review gives an overall life course approach or holistic approach to health looking at the environmental insults accumulated from conception to old age and different health outcomes.

### 4.2. Limitations of the Review

Many studies have established that to comprehensively evaluate the cause and progress of chronic diseases, there is the need to complement the genome with the exposome [29]. However, the acknowledgment of this fact and the analysis of environmental factors are being evaluated using questionnaires, geographic information, or a few targeted biomarkers hence lacking comprehensiveness or accuracy [30]. The exposome concept is defined as the totality of exposures from conception through to old age. This makes the birth cohort important and a core part of the study of the exposome. However, studies involving birth cohorts that use exposome as a framework are not common due to the long temporality making it a difficult point of research [31]. This is compounded by financial constraints. Such studies require prospective cohort study and participants must be followed for a long period of time. The long temporality makes the observation of such participants costly and difficult to sustain [32].

In addition, the exposome covers not only longitudinal environmental exposure from conception to old age but also overlapping domains of exposure namely, general external, the specific external, and the internal environments. General externals include the socio-economic environment (social capital, education level, neighborhood environment, and climatic factors), specific external include lifestyles, pollutant exposure, and occupations all these different aspects of exposome increase the complexity of the subject [33,34]. Internal factors include biological factors such as circulating hormones, metabolism, oxidative stress, inflammation, and gut microflora. These domain overlaps and making it difficult to assess them. Thus, measurement of exposome across the lifetime can lead to an overestimation of the real burden from the exposure by the possibility of measuring the effect it has on the individual more than once making it difficult to determine the real causes of diseases [16].

Heterogeneity of measurements and definitions of variables affect comparability, for instance the definition of vulnerability and susceptibility and how neighborhood characteristics are assessed such as quality. There have been different efforts from individual research teams to define and develop methodologies for measuring exposome. In the Human Genome Project, for instance, there were both national and international efforts for the sharing of data and coordination. This helped to minimize measurement error and also help in the development of the appropriate methodology. The National Institute of Environmental Health Sciences (NIEHS) has helped in the building of a centralized system for the provision of tools, data, and methods for exposome study, but there has been a limitation in terms of the number of proofs of the concept studies and there is no universal consensus in selecting what, when, and where to measure with regards to the concept of the exposome. Such heterogeneity makes it difficult for comparability [35].

Some of the studies in the review focused on the environmental factors and their influence on the mental health of children; however, these studies did not analyze how they influenced mental health outcomes such as depression. Consequentially these studies focused on the negative aspects of mental health without analyzing the mental health competence and the potential for modifications in the built environment to reduce health inequalities and social vulnerability.

To complement the limitations described earlier, our scoping review presents its own strengths and limitations. First, our search could suffer from study selection biases. Non-English publications of relevant articles may have been ignored. Furthermore, we cannot exclude the possibility that our review could be impacted by publication bias. Indeed, unpublished results (including grey literature and results not statistically significant, which are not available) may influence our findings

### 4.3. Recommendations for Futures Studies

Gender should be considered in the assessment of social vulnerability for various reasons. For instance, the genetic makeup of males and females affects their responses to environmental pollutants.

The occupation of the individuals during the time of their working life influences greatly the level of exposure to environmental pollutants. However, among the studies reviewed only one study assessed occupation. Research has shown that most of adult life is spent on the job hence much attention should be given to the occupational environment, especially for outdoor workers. The focus of the included studies was on residential and neighborhood-built environments which characterized more than 90% of the studies, the occupational environment should also be considered in future studies in light of gender roles.

Ethnicity/race should be considered in future studies when assessing social vulnerability and exposome. To better understand and help marginalized areas and populations their unique circumstances (culture) need to be considered. There also are many studies for instance that have shown differences in pregnancy outcomes among women from different race/ethnic backgrounds. For instance, non-Hispanic black women and non-Hispanic white women have different causes of death-related outcomes due to pregnancy, while deaths from pregnancy are a result of preeclampsia, for most non-Hispanic black women, deaths from pregnancy-related outcomes among non-Hispanic white women are due to mental health [36]. This will better tailor the needs of different groups and provide suitable interventions.

Breaking down the barriers between disciplines; research on the exposome calls upon many disciplines: biology, medicine, physics, chemistry, epidemiology, sociology, public health, etc. However, the dialogue between the quantitative sciences and the social sciences is not always self-evident. For instance, the importance of the gender concept in environmental health risk when included in research could strengthen methodologies used and study designs adopted [37]. An interdisciplinary approach that takes into account both air pollution and environmental injustice will ensure the overall health and safety of the generations to come. The three comprehensive overlapping domains of exposure: the general external, specific external, and internal environments all require different disciplines to fully comprehend the concept of the exposome.

The focus should be channeled towards children living in poor conditions to improve preventive measures by educating the parents to be aware of exposure impact on children population at an early age and most specifically among the poorer population.

In addition, there is the need to improve the pedagogy for parents that have difficulties to well understand the different messages (as several of them are more likely to have a low level of education). To do that, we need to implement a universal screening mechanism for social factors that identify children at risk from early exposure to harmful pollutants and link them to community resources to improve their health outcomes in later years, for example, children who are at risk of heart diseases can be identified during early screening to determine the family at risk or the vulnerable families and provide the needed assistance.

An information system to monitor child poverty can be developed in France to know where they live and to prioritize environmental interventions in areas with the highest level of environmental exposure. Interventions such as facilities to cater for the aged and most vulnerable populations can be implemented, through increasing and improving conditions in homes for the aged especially in marginalized areas, providing rehabilitations centers, and proper management and care for children or persons with disabilities or special needs children as a result of birth defects.

Lastly, community-level interventions should be employed in improving vulnerability at an early stage. The first point of call for children is their community where they grow up hence interventions through community level, with different strategies can aid in improving social vulnerability.

## 5. Conclusions

In conclusion, our work confirms that we are not all equal to face environmental nuisances. The poorest are more vulnerable to the health effect of environmental nuisances. It is known as a concept of social vulnerability, as defined by several authors [38,39]. For instance, in 2003, cutter et al. explained the complexity of the social vulnerability concept as a result of social inequalities, measure mainly at individual level, combined with place inequalities (defining the characteristics of communities and the built environment, and including environmental nuisance exposure) [38]. There is a growing interest of improving the level of understanding and of better defining the concept of vulnerability as recently summarized by Palacios, A. et al. in their recent review published in 2018 [19]. They reminded us that vulnerability is a multidimensional and a multifaced concept for which still today there is no clear definition consensus [19]. It is a call to continue the investigation in a methodological and practical ways to lead to studies based on similar framework background. The ongoing pandemic reminds us how unequal we are in facing this disease. Many recent studies found a disproportionate impact of COVID-19 according to sub-group of population. Rifat et al. revealed disparities in rates of case and mortality between the rural and urban counties. They also found an increase of cases and death rates of COVID-19 with the increase of many county characteristics as ethnicities and minorities, and the level of median income [40].

For building a healthy environment for all, and especially for the most vulnerable population, is a crucial issue if we want to design and implement measures for a greener and more equitable territory. The effect modification has been observed at different ages of life. Interventions should target in priority these high-risk populations. This review has identified gaps in the current research evidence on the importance of considering individual characteristics and behavior to fully understand vulnerability and exposome. Different disciplines are also required to fully explore the benefit of the exposome in understanding health outcomes. Further investigations are needed to formalize more precisely the concept of the social exposome and then communicate it.

## Figures and Tables

**Table 1:** Summary of the main characteristics of the selected studies.

Author and Date	Number of Studies	Population	Outcome	Location	Main Findings/Results	Limitations of the Study
Hibbert et al. (2019)	146 studies	Children	Asthma, cardiovascular diseases, chronic conditions (general), diabetes and cancer	United States, Canada, and Europe	1. Non-chemical stressors found in a child social environment can influence their health and wellbeing and influence their response to chemical exposure. 2. Adverse relationships exist between health and non-chemical stressors such as economic disadvantage, lower educational attainment, exposure to violence, adverse childhood experiences (ACE), stress, and urbanicity. 3. There is a salutary effect of non-chemical stressors such as exposure to or experience from green space and social support on health and well-being.	1. Identification of numerous inconsistencies in terminology leading to heterogeneity. 2. Lack of interdisciplinary research to bridge the gap between physical and social sciences which could strengthen the designs and methodologies. 3. The possibility of not capturing all the studies in this review.
Paterson et al. (2020)	15 studies	Older people, infants, pregnant women and children (People)	Cardiorespiratory disease diabetes, mental health psychosocial problems	Ireland	1. There is a heat-health vulnerability in Ireland and other temperate regions, especially among older people, chronically ill, infants, pregnant women, children, outdoor workers, socio-economically disadvantaged, urban dwellers thereby posing a public and occupational health challenge. 2. High and low temperatures were associated with adverse birth outcomes of preterm birth, low birth weight, and sometimes, stillbirth. 3. With heat and gender men are usually seen to be more vulnerable and suffer from heat than women.	Assessing mortality attributable to heat waves does not indicate the increased burden on the health sector from heat-related mortality.
Wong et al. (2017)	Not available	Childhood, Adulthood (People)	Congenital heart diseases	Asia, Americas, Europe	1. Stressors and toxic exposures during sensitive and critical periods of early development play critical roles in determining cardio metabolic risk over the life-course. 2. Interactions between maternal lifestyle factors that alter folate metabolism, such as obesity and carrier rates of certain genetic polymorphisms have been associated with increased risk of CHD. 3. Social antecedents play a significant role in conditioning disease burden and modulating outcomes of congenital heart disease.	N/A
Gelormino et al. (2015)	23 studies	People	Cardiovascular diseases, kidney diseases	America and Europe	1. The natural environment, social context, and behaviors are all connected, partially or totally, to one of the following components: density and availability of public spaces and may influence individual health.	1. There is heterogeneity among studies in the measurement of socio-economic status, the level of geographic aggregate considered, and the confounders accounted for. 2. Some of the studies used did not have the main objective of the research. 3. There is a lack of depth due to the absence of multi-disciplinarity. 4. The external validity of these studies is questionable, due to the significant effect of context, local research.
Benmarhnia et al. (2015)	61 studies	People	Mortality	Asia, Americas, Europe	1. The strongest evidence of heat-related vulnerability was for the elderly ages >65 and >75 years and low SES groups (at the individual level), they were more vulnerable than their respective counterparts using the pooled estimates.	1. Heterogeneity among studies, due to contrast definitions and other factors complicates the interpretation of a single summary estimate. 2. Several studies were excluded because the statistical heterogeneity test could not be performed. 3. The definition of vulnerability in this review was an epidemiological definition; however, vulnerability encompasses a social dimension. 4. In the literature reviewed in this article, vulnerability factors were considered separately but there are several modifying factors that might interact synergistically in the heat-related mortality relationship.
Mathiarasan S et al. (2021)	Not available	Children	Neuropsychological health problems, respiratory diseases, sleep disorders and mental health issues.	Not available	1. Air pollution disproportionately affects marginalized populations of lower socioeconomic status, children of lower socioeconomic status and are likely to be more exposed to both indoor and outdoor air pollution.	N/A
Alderton et al. (2019)	14 studies	Children (<8 years) and (>8 years)	Mental health	Europe and United States	Neighborhood built environment may be important for reducing mental health difficulties and increasing mental health competence among children.	There are gaps in the evidence hence there is the need to examine associations with positive aspects of mental health (mental health competence), the role of understudied neighborhood attributes like social infrastructure and service quality, and also different associations between the neighborhood-built environment and mental health in early years and the potential for modifications in the built environment to reduce health inequalities.
Fuller et al. (2017)	30 articles	People	Cardiovascular disease and mortality, hypertension, Ischemic heart disease, myocardial infarction	America, Europe, Asia, and Canada	1. Adult never/former smokers are at a higher risk of incident asthma due to air pollution. 2. Children without atopy and children from low socio-economic status are at a higher risk of incident asthma due to air pollution.	1. The use of identical measures across studies may not be appropriate across the board, for example, measures that capture SEP well for one population may not do so for another. 2. Area level measures of Socioeconomic position, proximal and distal factors that accumulate across the life course could modify air pollution associations with health and should be taken into consideration. 3. Large sample size is needed. 4. A full systematic review and meta-analysis were outside the scope of this review due to the heterogeneity of the exposure and outcome measures used.
Burte et al. (2016)	25 studies	Children and Adult (People)	Asthma	Canada, USA, Japan, Sweden	1. Never/former smoker adults seem to be more susceptible to air pollution in relation to incident asthma. 2. Children without atopy seem to have a higher risk of incident asthma due to air pollution as well as children with low SES. 3. None of the studies included in the review was explicitly designed to assess the susceptibility factors concerning the associations between air pollution and incident asthma.	N/A
Schule et al. (2015)	33 studies	People	Depressive symptoms.	USA, Canada, Australia, New Zealand and Western European countries	1. Independent association between characteristics of neighborhood SEP or the built environment and individual health outcomes or health-related behaviors. 2. Low neighborhood SES was independently associated with poor health, such as increased mortality r poor self-rated health, depressive symptoms, low birth weight, or cardiovascular risk factors. 3. The built environment has a significant impact on health outcomes.	N/A
Erickson et al. (2014)	Not available	Pregnant women	Adverse pregnancy outcomes (early/recurrent miscarriages, hypertension, preeclampsia, fetal growth restriction, placenta abruption, pre-labour rapture of the fetal membranes (PROM) and spontaneous preterm labour), obesity, diabetes, cardiovascular and reproductive diseases.	Not available	Socioeconomic disparities are known to confound the environmental exposure effects, however, they may also act as potential effect modifiers given their overlapping etiological mechanisms with PM 2.5 exposure.	

## Data Availability

All data are available from pubmed website.
